# Three mitochondrial lineages and no Atlantic-Mediterranean barrier for the bogue Boops boops across its widespread distribution

**DOI:** 10.1038/s41598-022-26651-8

**Published:** 2022-12-21

**Authors:** Regina L. Cunha, Abderraouf Ben Faleh, Sara Francisco, Radek Šanda, Jasna Vukić, Luana Corona, Mamadou Dia, Igor Glavičić, Abderrahmane Kassar, Rita Castilho, Joana I. Robalo

**Affiliations:** 1 Centre of Marine Sciences-CCMAR/CIMAR LA, Campus de Gambelas, 8005-139 Faro, Portugal; 2 University of Algarve, Campus de Gambelas, 8005-139 Faro, Portugal; 3 Laboratory of Ecology, Biology and Physiology of Aquatic Organisms (LR/18/ES/41), Faculty of Sciences of Tunis, University of Tunis El Manar, Tunis, Tunisia; 4 MARE-Marine and Environmental Sciences Centre/ARNET-Aquatic Research Network, Ispa-Instituto Universitário de Ciências Psicológicas, Sociais e da Vida, Lisbon, Portugal; 5 Department of Zoology, National Museum, Vaclavske Namesti 68, 115 79 Prague, Czech Republic; 6 Department of Ecology, Charles University, Vinicna 7, 128 44, Prague, Czech Republic; 7 Institut Mauritanien de Recherches Océanographiques et des Pêches, Nouadhibou, Mauritania; 8 Department of Marine Studies, University of Split, Split, Croatia; 9 Département des Ressources Vivantes‚ Ecole Nationale Supérieure des Sciences de la Mer et de l’Aménagement du Littoral - ENSSMAL, Algiers, Algeria

**Keywords:** Haplotypes, Population genetics

## Abstract

Marine species exhibiting wide distributional ranges are frequently subdivided into discrete genetic units over limited spatial scales. This is often due to specific life-history traits or oceanographic barriers that prevent gene flow. Fine-scale sampling studies revealed distinct phylogeographic patterns in the northeastern Atlantic and the Mediterranean, ranging from panmixia to noticeable population genetic structure. Here, we used mitochondrial sequence data to analyse connectivity in the bogue *Boops boops* throughout most of its widespread distribution. Our results identified the existence of three clades, one comprising specimens from the Azores and eastern Atlantic/Mediterranean, another with individuals from the Canary Islands, Madeira and Cape Verde archipelagos, and the third with samples from Mauritania only. One of the branches of the northern subtropical gyre (Azores Current) that drifts towards the Gulf of Cádiz promotes a closer connection between the Azores, southern Portugal and the Mediterranean *B. boops* populations. The Almería-Oran Front, widely recognised as an oceanographic barrier for many organisms to cross the Atlantic-Mediterranean divide, does not seem to affect the dispersal of this benthopelagic species. The southward movement of the Cape Verde Frontal Zone during the winter, combined with the relatively short duration of the pelagic larval stage of *B. boops,* may be potential factors for preventing the connectivity between the Atlantic oceanic archipelagos and Mauritania shaping the genetic signature of this species.

## Introduction

The existence of population genetic structure in the marine realm is often considered paradoxical given the apparent absence of physical barriers^1^. Nonetheless, an increasing number of studies reported significant population diversification over limited spatial scales in species exhibiting large geographic ranges due to various processes^2^. Among those, life-history traits associated with limited dispersal abilities, such as determined by short pelagic larval durations (PLD)^3^ or oceanographic barriers that avert gene flow^4,5^, may play an essential role in shaping the genetic structure of the species. Studies performing meta-analyses and fine-scale sampling revealed the existence of distinct phylogeographic patterns in the northeastern Atlantic and Mediterranean. These patterns range from fish species displaying noticeable population structure^6^ to others exhibiting panmixia throughout their geographic ranges^7^. Historical events and oceanographic patterns generate species-specific responses^8^.

The bogue *Boops boops* (Linnaeus, 1758) (family Sparidae) is a gregarious, benthopelagic fish species inhabiting depths between 0 and 350 m^9^. This coastal species is broadly distributed in the Mediterranean and eastern Atlantic (from Norway to Angola), including the oceanic archipelagos at these latitudes^10^. It represents a vital fishery resource, particularly in the eastern Mediterranean^11^, inhabiting diverse habitats, including rocky substrates, sandy bare seabeds, seaweeds and seagrass meadows^12^. *Boops boops* have a PLD of 16 to 18 days and exhibit a vertical pattern of larval assemblages: smaller larvae occur at the surface while larger individuals are more frequently found at the bottom^13^. During the summer, groups of adult individuals approach shallow coastal waters, while in winter migrate to waters deeper than 100 m^14^. Until now, studies on the bogue have focused on biological aspects^15–17^ or on the species' impact on fish community assemblages and aquaculture^18,19^. Defining the spatial structure of intraspecific genetic diversity is paramount for fisheries stock assessment^20^ and understanding how oceanographic features affect population connectivity.

**Figure 1:**
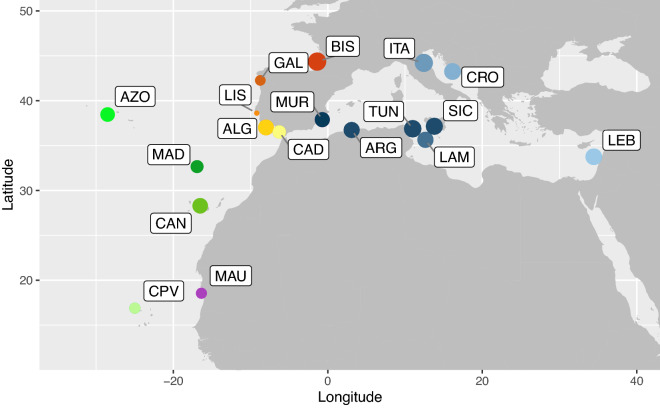
Sampling sites across the distributional range of *Boops boops* in the Northeastern Atlantic and the Mediterranean. Location codes in Table 1. Circles size proportional to samples sizes. Figure generated using the worldHires (http://CRAN.R-project.org/package=mapdata) function implemented in R language^24^. URL https://www.R-project.org/) (version 3.3.1), which uses publicly available coastline coordinates from the NOAA National Geophysical Data Center (http://www.ngdc.noaa.gov/mgg/shorelines/shorelines.html).

In the present study, we used the mitochondrial control region (D-loop) to infer genetic connectivity in *B. boops* throughout most of its geographic range in the eastern Atlantic, from the Bay of Biscay to Mauritania, including the archipelagos of Madeira, Azores, Canary Islands and Cape Verde (Fig. 1). The species is distributed across some oceanographic barriers including, e.g. the Almería-Oran Front in the Atlantic-Mediterranean transition zone^21^. To analyse the putative effect of this oceanographic barrier on the genetic structure of the species, we also included several sampling locations in the Mediterranean (Algeria, Tunisia, Lebanon, Croatia, Spain and Italy).

## Materials and methods

### Sampling collection

Collections of *B. boops* tissue (n = 335) were obtained from several locations across the distributional range of the species in the Atlantic and Mediterranean: Bouharoun, Algeria (ARG); Azores, Portugal (AZO); Bay of Biscay, Spain (BIS); Cádiz, Spain (CAD); Canary Islands, Spain (CAN); Mindelo, Cape Verde (CPV); Brač Island, Croatia (CRO); Galícia, Spain (GAL); Cesenatico, Italy (ITA); Lampedusa, Italy (LAM); Tyre and Tripoli, Lebanon (LEB); Lisbon and Tróia, Portugal (LIS); Madeira Island, Portugal (MAD); Nouadhbou, Mauritania (MAU); Murcia, Spain (MUR); Ria Formosa, Portugal (ALG); Sicily, Italy (SIC); Bizerte and Kélibia, Tunisia (TUN) (Fig. 1 and Table 1). Specimens were collected by local fishermen or from local markets. After asserting the species identification for each individual, fins were clipped and preserved in 96% ethanol.

**Table 1:** Sample locations, sizes and summary statistics for the mitochondrial control region of *Boops boops*.

Region	Country	Site	Code	Lat	Lon	N	NH	H ± s.d	π ± s.d
Atlantic	Spain	Bay of biscay	BIS	44.36	− 1.38	30	30	1.000 ± –	0.031 ± 0.016
Atlantic	Spain	Galícia	GAL	42.38	− 8.57	9	9	1.000 ± –	0.033 ± 0.019
Atlantic	Portugal	Lisbon	LIS	38.69	− 9.20	3	3	1.000 ± –	0.042 ± 0.032
Atlantic	Portugal	Azores	AZO	38.56	− 16.92	20	19	0.995 ± 0.013	0.030 ± 0.016
Atlantic	Portugal	Algarve	ALG	37.02	− 8.00	22	22	1.000 ± –	0.031 ± 0.016
Atlantic	Spain	Cádiz	CAD	36.52	− 6.28	15	15	1.000 ± –	0.031 ± 0.017
Atlantic	Portugal	Madeira	MAD	32.66	− 16.93	14	12	0.978 ± 0.027	0.016 ± 0.009
Atlantic	Spain	Canaries	CAN	28.29	− 16.52	21	15	0.924 ± 0.049	0.013 ± 0.008
Atlantic	Mauritania	Nouadhibou	MAU	18.55	− 16.36	10	9	0.978 ± 0.043	0.019 ± 0.011
Atlantic	Cape verde	Mindelo	CPV	16.88	− 24.98	10	10	1.000 ± –	0.018 ± 0.010
Mediterranean	Spain	Murcia	MUR	37.89	− 0.71	20	20	1.000 ± –	0.031 ± 0.016
Mediterranean	Algeria	Bouharoun	ARG	36.64	2.63	19	17	0.982 ± 0.022	0.014 ± 0.008
Mediterranean	Tunisia	Bizerte and kélibia	TUN	36.88	10.99	23	22	0.996 ± 0.011	0.032 ± 0.017
Mediterranean	Italy	Cesenatico	ITA	44.21	12.43	25	23	0.990 ± 0.014	0.027 ± 0.014
Mediterranean	Italy	Lampedusa	LAM	35.64	12.64	23	22	0.996 ± 0.011	0.030 ± 0.016
Mediterranean	Italy	Sicily	SIC	37.15	13.79	22	16	0.974 ± 0.015	0.025 ± 0.013
Mediterranean	Croacia	Brač Island	CRO	43.25	16.15	25	23	0.990 ± 0.014	0.030 ± 0.016
Mediterranean	Lebanon	Tyre/tripoli	LEB	33.76	34.43	24	23	0.996 ± 0.010	0.027 ± 0.014
Total				–	–	335	300	0.998 ± 0.001	0.049 ± 0.024

*Lat* latitude, *Lon* longitude, *N* number of individuals, *NH* number of haplotypes, *h* haplotype diversity, **π** nucleotide diversity.

### DNA extraction, amplification and sequencing

Total genomic DNA was extracted with the REDExtract-N-Amp Kit (Sigma-Aldrich) following the manufacturer's instructions. The mitochondrial control region (D-loop) was amplified in a Bio-Rad Mycycler thermal cycler, using the primers L-pro1 and H-DL1^22^. The PCR protocol was performed in a 20 μl total reaction volume with 10 μl of REDExtract-N-ampl PCR mix (Sigma-Aldrich), 0.8 μl of each primer (10 μM), 4.4 μl of Sigma water and 4 μl of template DNA. PCR conditions were the following: initial denaturation at 94 °C for 2 min., followed by 35 cycles (denaturation at 94 °C for 30 s., annealing at 55 °C for 45 s., and extension at 72 °C for 1 min.) and a final extension at 72 °C for 10 min. The same primers were used for the sequencing reaction, and the PCR products were purified and sequenced in STABVIDA (http://www.stabvida.net/). Chromatograms were edited with Codon Code Aligner (Codon Code Corporation, http://www.codoncode.com/index.htm), and sequences were aligned with Clustal X 2.1^23^. All sequences were deposited in GenBank (Accession numbers ON605268-ON605602).

### Data analysis

The R^24^ packages *haplotypes*^25^ and *pegas*^26^ were used in RStudio^27^ to estimate standard descriptive measures of genetic diversity in *B. boops*, including the number of haplotypes and private haplotypes, haplotype (*h*,^28^ and nucleotide (π,^28^ diversities. The genetic structure among all locations was assessed by AMOVA^29^ using Arlequin 3.5.2.2^30^ with several site group arrangements. The genetic group constitution was assessed by Φct, while the pairwise differentiation between all locations was assessed by Φst. We used Φst and Φct because, contrary to its analogues *F*st and *F*ct, these parameters consider the genetic differences between haplotypes. The significance of each pairwise comparison was tested with 10,000 random replicates, and multiple comparisons were corrected using the false discovery rate^31^ to calculate q-values (q-values < 0.05 were set as the significant threshold).

The PopART software^32^ (https://github.com/jessicawleigh/popart-current.git) was used to build a haplotype network based on the Minimum Spanning algorithm^33,34^. To further visualize relationships between haplotypes at population level, we used the software Network v. 10.2.0.0^34^ to build a Median Joining network (https://www.fluxus-engineering.com/sharenet.htm)*.* Pairwise nucleotide distances (p-distance, 500 bootstrap replicates) within and between *B. boops* clusters defined by AMOVA were computed in MEGA7^35^. We used the pheatmap function of the R package with the same name^36^ (https://cran.r-project.org/web/packages/pheatmap/index.html) to obtain a heatmap, which is a graphical representation of the individuals in a matrix to visualize the similarity and dissimilarity among individuals with colors, and simultaneously generate a dendogram to better visualise the clusters.

We used ABGD (Automatic Barcode Gap Discovery)^37^ (https://bioinfo.mnhn.fr/abi/public/abgd/abgdweb.html) to analyse putative cryptic diversity within *B. boops*. ABGD is based on identifying the barcode gap, i.e., a larger divergence among individuals of different species than among conspecific individuals. We selected the Jukes-Cantor model, the X-value of 1.5 (minimum relative barcoding gap width), and prior intraspecific divergences ranging from Pmin = 0.001 to Pmax = 0.1 to run the barcode gap analysis.

### Ethics

No experiments on live vertebrates were performed in this study.

## Results

A 403-fragment of the D-loop region produced 300 haplotypes in 335 *B. boops* individuals collected from 18 locations throughout the Western Atlantic and Mediterranean distribution. Haplotype diversities were high, ranging between 0.924 (in samples from the Canary Islands) and 1.000 in several locations of the Atlantic and Mediterranean (average = 0.989; all samples combined = 0.998). Nucleotide diversities varied between 0.013 (in samples from the Canary Islands) and 0.042 (in samples from Lisbon) (average = 0.027; all samples combined = 0.049) (see Table 1 for further details). Four indels were observed, and 281 (94%) haplotypes occurred as singletons. The most abundant haplotype was shared among 10 individuals from Algarve (1), Croatia (3), Italy (3), Lampedusa (1) and Lebanon (2), while the second most abundant was shared between Canary Islands (6) and Madeira (2). We also detected five shared haplotypes between the Canary Islands and Madeira.

Several site group arrangements were tried with the AMOVA. The three-group structure revealed (1) the Mediterranean and eastern Atlantic, including the Azores (hereafter designated as ATL/MED), (2) Canary Islands, Madeira and Cape Verde archipelagos (hereafter designated as ILS) and (3) Mauritania (hereafter designated as MAU) is supported by the highest highly significant Φ*ct-*AMOVA (0.76, p < 0.001) of the several groups tried (results not shown). The Φst-based values (Table 2) revealed differences compatible with this three-group structure.

**Table 2:** Pairwise differentiation for the mitochondrial control region of *Boops boops* based on Φst values (below diagonal) and significant q-values (above diagonal).

Φst/q-values	Algarve	Algeria	Azores	Biscay	Cadiz	Croatia	Galicia	Italy	Lampedusa	Lebanon	Lisbon	Murcia	Sicily	Tunisia	Canary Is.	Cape Verde	Madeira	Mauritania
Algarve	–	0.001	0.041	0.903	0.285	0.545	0.353	0.458	0.181	0.726	0.196	0.460	0.586	0.568	**0.000**	**0.000**	**0.000**	**0.000**
Algeria	0.058	–	0.001	0.006	0.065	0.021	**0.000**	0.001	0.001	0.005	0.041	0.010	0.002	**0.000**	**0.000**	**0.000**	**0.000**	**0.000**
Azores	0.024	0.056	–	0.009	0.036	0.042	0.017	0.005	0.016	0.002	0.112	0.089	0.006	0.003	**0.000**	**0.000**	**0.000**	**0.000**
Biscay	0.000	0.034	0.030	–	0.367	0.654	0.148	0.174	0.556	0.404	0.326	0.609	0.486	0.810	**0.000**	**0.000**	**0.000**	**0.000**
Cádiz	0.006	0.025	0.025	0.002	–	0.466	0.201	0.107	0.041	0.112	0.417	0.302	0.105	0.463	**0.000**	**0.000**	**0.000**	**0.000**
Croatia	0.000	0.030	0.021	0.000	0.000	–	0.046	0.612	0.620	0.504	0.152	0.058	0.390	0.235	**0.000**	**0.000**	**0.000**	**0.000**
Galícia	0.005	**0.132**	0.050	0.017	0.016	0.036	–	0.005	0.048	0.205	0.402	0.825	0.091	0.205	**0.000**	**0.000**	0.000	**0.000**
Italy	0.000	0.051	0.043	0.008	0.016	0.000	0.061	–	0.518	0.636	0.100	0.054	0.861	0.323	**0.000**	**0.000**	**0.000**	**0.000**
Lampedusa	0.010	0.055	0.031	0.000	0.024	0.000	0.037	0.000	–	0.553	0.168	0.226	0.695	0.768	**0.000**	**0.000**	**0.000**	**0.000**
Lebanon	0.000	0.043	0.045	0.001	0.016	0.000	0.015	0.000	0.000	–	0.244	0.360	0.963	0.350	**0.000**	**0.000**	**0.000**	**0.000**
Lisbon	0.046	0.187	0.070	0.016	0.007	0.051	0.010	0.072	0.052	0.035	–	0.252	0.128	0.287	0.001	0.002	0.001	0.004
Murcia	0.000	0.039	0.017	0.000	0.006	0.018	0.000	0.019	0.008	0.003	0.029	–	0.376	0.389	**0.000**	**0.000**	**0.000**	**0.000**
Sicily	0.000	0.052	0.037	0.000	0.018	0.002	0.029	0.000	0.000	0.000	0.068	0.003	–	0.649	**0.000**	**0.000**	**0.000**	**0.000**
Tunisia	0.000	**0.060**	0.045	0.000	0.000	0.006	0.014	0.003	0.000	0.002	0.020	0.002	0.000	–	**0.000**	**0.000**	**0.000**	**0.000**
Canary	**0.794**	**0.877**	**0.808**	**0.785**	**0.810**	**0.796**	**0.819**	**0.809**	**0.798**	**0.811**	0.850	**0.802**	**0.821**	**0.785**	–	0.232	0.596	**0.000**
Cape Verde	**0.755**	**0.862**	**0.771**	**0.751**	**0.766**	**0.760**	**0.768**	**0.778**	**0.762**	**0.779**	0.798	**0.763**	**0.790**	**0.745**	0.016	–	0.452	**0.000**
Madeira	**0.765**	**0.863**	**0.779**	**0.759**	**0.778**	**0.770**	0.784	**0.785**	**0.771**	**0.786**	0.815	**0.773**	**0.797**	**0.756**	0.000	0.000	–	**0.000**
Mauritania	**0.744**	**0.849**	**0.760**	**0.739**	**0.750**	**0.750**	**0.757**	**0.765**	**0.751**	**0.768**	0.777	**0.748**	**0.779**	**0.733**	**0.874**	**0.849**	**0.854**	–

Values were corrected using the false discovery rate based on the Benjamini–Hochberg method^1^ (< 0.05 was set as the significant threshold).

Significant Φst and q-values are presented in bold.

**Figure 2:**
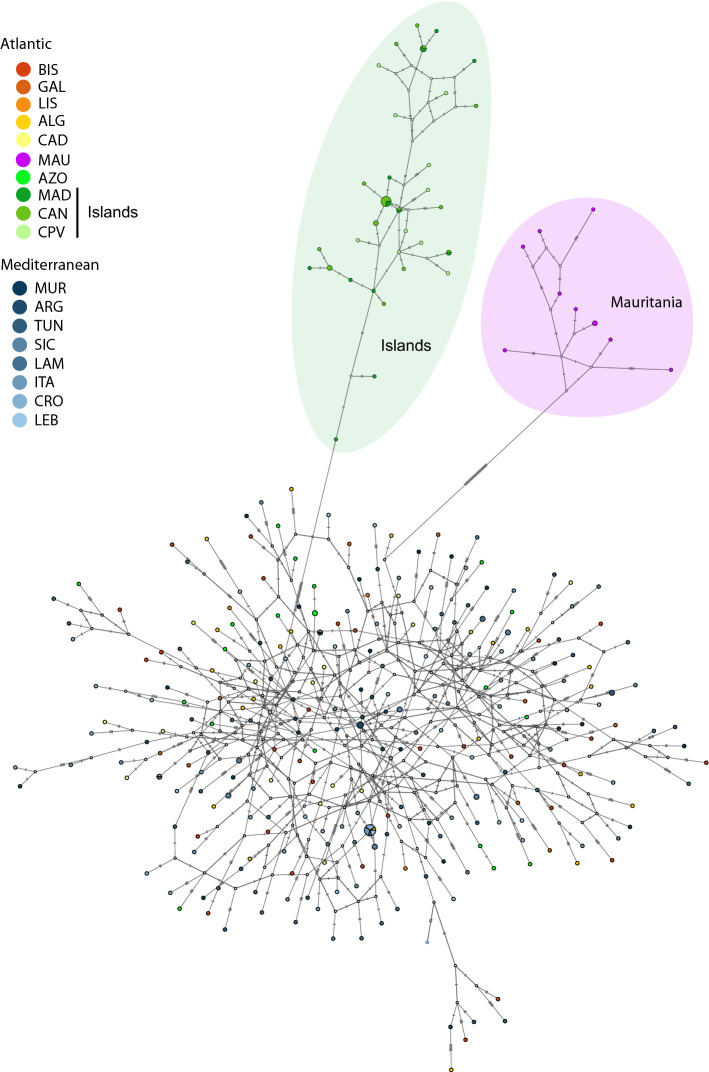
Haplotype network for the mitochondrial control region of *Boops boops* showing the existence of three main groups. The green shade highlights the group including samples from the Canary Islands, Cape Verde and Madeira Islands. The purple shade represents the clade corresponding to the group including individuals from Mauritania. The third group corresponds to samples from the Atlantic/Mediterranean and the Azores. Colours refer to sampling locations. The area of the circles is proportional to each haplotype frequency. In the case where haplotypes are shared among sampling locations, filling is proportional to the frequency of the haplotype in each sampling location.

**Figure 3:**
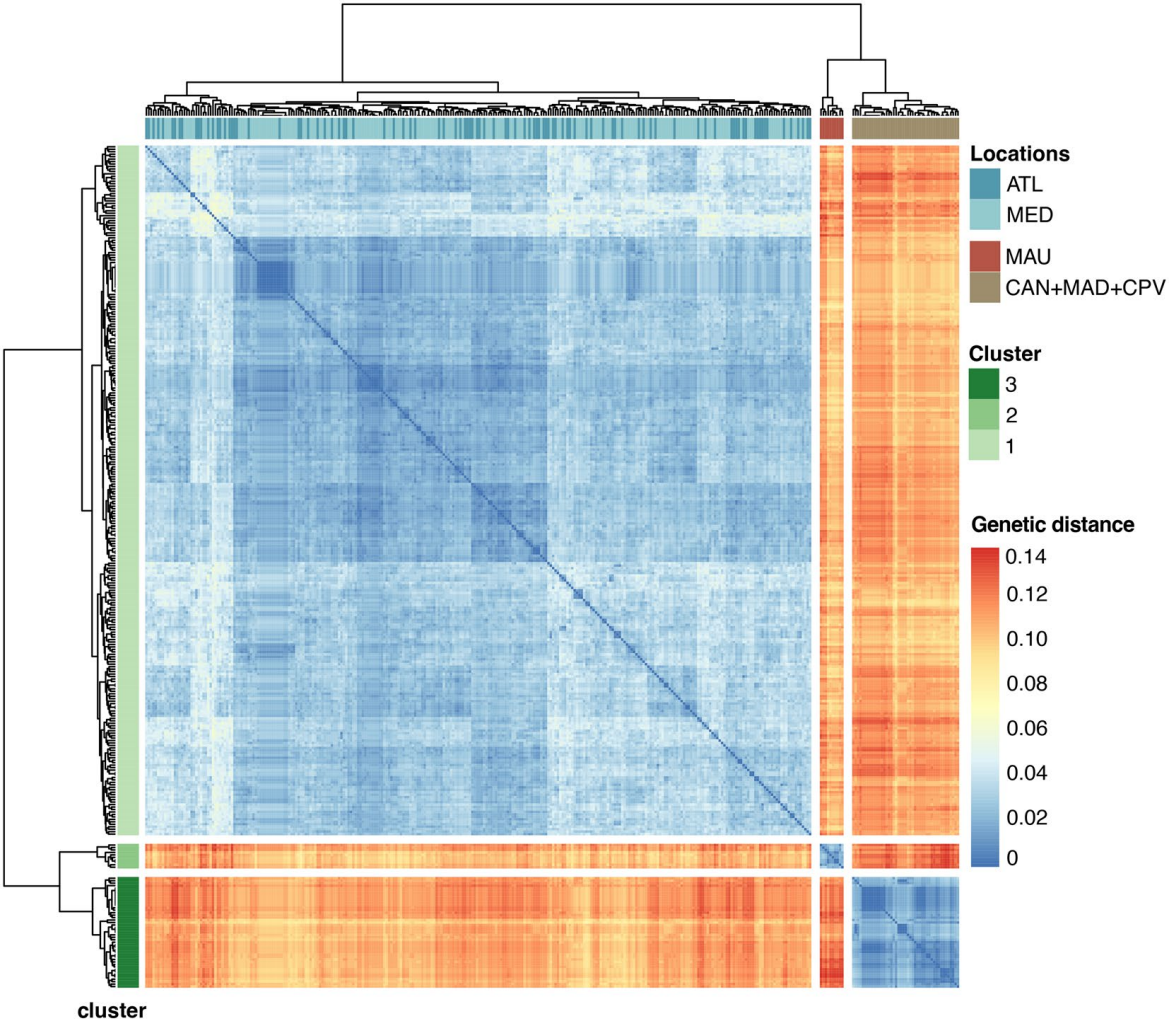
Heatmap and dendrogram based on pairwise distances based on Kimura (1980) for the mitochondrial control region of *Boops boops*. The heatmap and the associated dendrogram were generated using the pheatmap R-package (https://cran.r-project.org/web/packages/pheatmap/index.html).

Both haplotype network building algorithms, Minimum Spanning (Fig. 2) and Median Joining (Supplementary material S1) identified the three distinct clades (ATL/MED, ISL and MAU). The clade with samples from Mauritania was separated from the ATL/MED by 25 mutational steps. The clade, including individuals from the Atlantic islands (ISL), was also separated by 25 mutational steps from ATL/MED. The hierarchical clustering visualised in the heatmap also shows the existence of those three clusters ATL/MED, ISL—and MAU (Fig. 3). Within-group estimates of average divergence were the following: MAU—2% ± 0.4%; ISL—1.5% ± 0.3% and ATL/MED—2.9% ± 0.4%. Net divergences were the following: between ATL/MED vs. MAU was 7.5% ± 1.2%; between ATL/MED vs. IS L was 7.9% ± 1.2%; between MAU vs. ISL was 9.6% ± 1.4%. ABGD results identified three Evolutionary Significant Units (ESU) within *B. boops* coinciding with the AMOVA results.

## Discussion

The main finding of the present study is the identification of genetic structure in a benthopelagic short-sized species that disperses over large distances. We found evidence for the existence of three genetic clusters within the bogue *B. boops*, one comprising specimens from the Azores and eastern Atlantic/Mediterranean, another with individuals from the eastern Atlantic archipelagos (Canary Islands, Madeira and Cape Verde), and the third with samples from Mauritania (Figs. 2 and 3). The genetic divergence between haplogroups (between 7.5% and 9.6%) is compatible with differences at the genus level found in several fish species^38^. Further molecular and morphological analyses are required to confirm the existence of cryptic lineages within *B. boops*.

Identifying processes generating population differentiation within a species is crucial to understanding its evolutionary dynamics. The interplay between life-history traits (e.g. adult swimming abilities, PLD or larval behaviour) and oceanographic features often plays an important role in the dispersal range of a species^39^. For instance, the shanny *Lipophrys pholis* occurs throughout the eastern Atlantic and Mediterranean rocky shores showing no signal of genetic structure along its distribution^40^. The dispersal of *L. pholis* occurs exclusively during the 29 days that last its pelagic larval phase, which allows widespread gene flow between distant locations^41^. The heatmap from Fig. 3 shows that *B. boops* specimens from the Atlantic and Mediterranean share haplotypes. These results indicate that the Almería-Oran Front, an oceanographic barrier known to prevent gene flow between populations from the Atlantic and Mediterranean in more than 50 marine species^42^, does not represent a barrier to dispersal in *B. boops.* The lack of genetic structure between these two basins was also identified in another species of Sparidae (*Sarpa salpa*), in which the strong swimming abilities of the larvae seem to promote connectivity among populations^43^. Also, it has been proposed that the Portuguese coast could be considered a sort of "Mediterranean appendix, rather than a biogeographic bridge"^44^, which is supported by our results.

From June to October, the Cape Verde Frontal Zone migrates eastward from Guinea-Bissau and Cape Verde waters to the Cape Blanc headland (Mauritania), while during the rest of the year it moves southward^45^. If the dispersal of *B. boops* in this area depended on the adults' movement, we would expect a closer relationship between Cape Verde and Mauritanian populations. Instead, the genetic analyses revealed that specimens from Cape Verde, Madeira and Canary Islands grouped into a cluster different from the one including the Mauritania samples (Figs. 2 and 3). *Boops boops* is a demersal coastal species wherein large shoals of adults migrate into deeper waters in the spawning season between January and May, peaking in February,^15^. The southward direction of the Cape Verde Frontal Zone during the winter and the relatively short PLD of *B. boops* between 16 and 18 days,^13^, must prevent the movement of the larvae from Cape Verde to Mauritania. The Mauritanian population persists without admixture of individuals from the eastern Atlantic archipelagos, probably due to the gregarious nature of the adults that, during summer, aggregate in large shoals in shallow waters to feed in one of the most productive marine regions of the world's oceans, the Mauritania upwelling^46^. Net divergences between groups are larger between the eastern Atlantic archipelagos vs. Mauritania (0.096 ± 0.014) than between Atlantic/Mediterranean vs. Mauritania (0.075 ± 0.012). These results further support the low levels of connectivity between the eastern Atlantic islands and the Mauritanian population.

There are several examples of fish species exhibiting distinct genetic patterns within the same geographic range^5,8^. For instance, the sand-smelt (*Atherina presbyter*) populations from the Azores show a closer relationship with the eastern Atlantic archipelagos (Canary Islands and Madeira) and are markedly distinct from specimens of the Mediterranean and southern European Atlantic shores^47^. This pattern may have resulted from the colonisation of the Azores by the Canary Islands and Madeira acting as stepping stones and glacial refugia for fish coming from West Africa^47^. *Boops boops* haplotypes from southern Portugal and the Mediterranean are closely related to samples from the Azores. Yet, it is impossible to quantitatively infer the direction of the dispersal because there are few shared haplotypes and none with the Azores. Nonetheless, a West–East direction is expected due to a branch of the northern subtropical gyre (Azores Current) that drifts towards the Gulf of Cádiz. This current promotes the displacement of larvae and adults from the Azores to southern Portugal and into the Mediterranean, which is consistent with our pattern (Fig. 2).

Although based on a single-marker approach, our results are clear and reflect a comprehensive sampling coverage with a suitable number of individuals. The marker choice, frequently used in traditional phylogeographical approaches, allows comparisons with other species across the same geographical area, preventing biases resulting from different evolutionary rates.

## Conclusions

Identifying the processes that generate population differentiation in a species with a broad distribution is pivotal to understand its evolutionary dynamics. Here we show the existence of three clades within the bogue *Boops boops* that most likely correspond to distinct populations. One comprises samples from the Azores, eastern Atlantic and Mediterranean. Another includes samples from Madeira, Canary Islands and Cape Verde archipelagos. The third cluster is composed of specimens from Mauritania only. The Almería-Oran Front, a known oceanographic barrier for more than 50 species, does not prevent gene flow through the Atlantic-Mediterranean divide. The southward direction of the Cape Verde Frontal Zone during the winter (spawning season of *B. boops*) combined with the relatively short PLD of this species averts the genetic admixture between populations from Mauritania and the neighbour eastern Atlantic archipelagos. The gregarious nature of the adults that remain in shallow waters during the summer to feed in the highly productive marine system of the Mauritanian upwelling, further prevents connectivity with nearby oceanic islands' populations. The observed genetic discontinuities do not conform to an isolation-by-distance model but instead reflect the role of specific life-history traits and local oceanographic features.

## Supplementary Information

Supplementary Information.

## Data Availability

All sequences produced in this study were deposited in GenBank under the accession numbers ON605268-ON605602.
