# Supplementary material for: Three mitochondrial lineages and no Atlantic-Mediterranean barrier for the bogue Boops boops across its widespread distribution
Source: Sci Rep. 2022 Dec 21;12:22124. doi: 10.1038/s41598-022-26651-8 (PMC9772343; doi:10.1038/s41598-022-26651-8)

Supplementary material_S1. Medium joining haplotype network for the mitochondrial control region of *Boops boops* showing the existence of three main groups. The green circle highlights the group including samples from the Canary Islands, Cape Verde archipelago and Madeira Islands. The purple circle includes the clade corresponding to samples from Mauritania only. The third group corresponds to samples from the Atlantic/Mediterranean and the Azores. Dashes on the lines connecting the three groups represent mutational steps. The figure was obtained with the software network (https://www.fluxus-engineering.com/sharenet.htm).

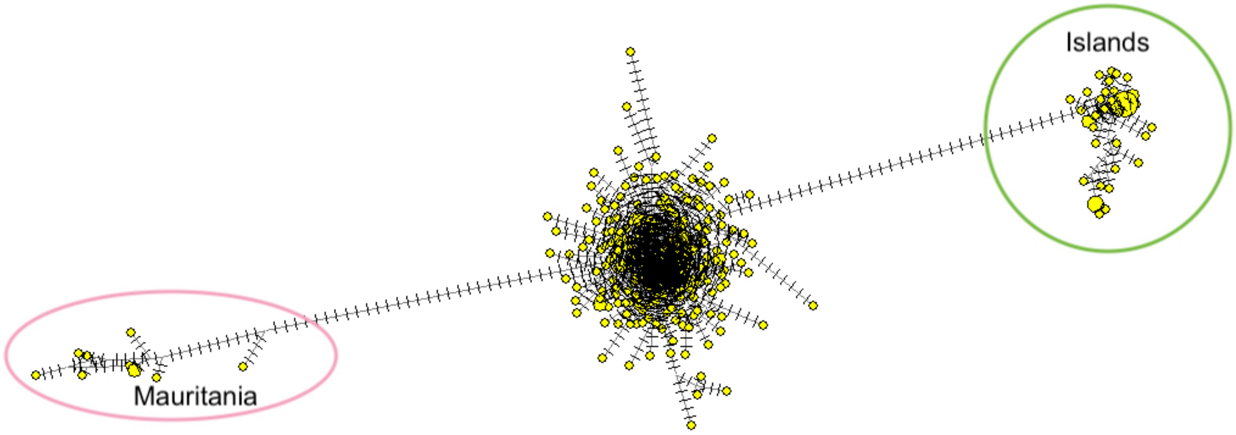

Supplement: Supplementary file 1 — Supplementary Information. [file 41598_2022_26651_MOESM1_ESM.pdf]
